# Morphological variation in a secondary contact between divergent lineages of brown trout (*Salmo trutta*) from the Iberian Peninsula

**DOI:** 10.1590/S1415-47572009005000014

**Published:** 2009-01-16

**Authors:** Miguel Hermida, Eduardo San Miguel, Carmen Bouza, Jaime Castro, Paulino Martínez

**Affiliations:** Departamento de Genética, Facultad Veterinaria, Universidad de Santiago de Compostela, LugoSpain

**Keywords:** brown trout, secondary contact, morphometric, meristic, multivariate analysis

## Abstract

The aim of this study was to analyze the morphological variation of brown trout (*Salmo trutta*) in the Duero basin, an Atlantic river basin in the Iberian Peninsula, where a spatial segregation of two divergent lineages was previously reported, based on isozyme, microsatellite and mtDNA data. In these studies, two divergent pure regions (Pisuerga and Lower-course) and several hybrid populations between them were identified. Morphological variation was evaluated in 11 populations representative of the genetic differentiation previously observed in the Duero basin, using multivariate analysis on 12 morphometric and 4 meristic traits. A large differentiation between populations was observed (interpopulation component of variance: 41.8%), similar to that previously detected with allozymes and microsatellites. Morphometric differentiation was also reflected by the high classification success of pure and hybrid individuals to their respective populations, using multivariate discriminant functions (94.1% and 79.0%, respectively). All multivariate and clustering analyses performed demonstrated a strong differentiation between the pure regions. The hybrid populations, though showing large differentiation among them, evidenced an intermediate position between the pure samples. Head and body shape traits were the most discriminant among the morphometric characters, while pectoral rays and gillrakers were the most discriminant among the meristic traits. These results confirmed the high divergence of the brown trout from the Duero basin and suggest some traits on which selection could be acting to explain the spatial segregation observed.

## Introduction

The brown trout (*Salmo trutta*) is one of the most well researched European fish. It is also one of the vertebrate species with highest genetic subdivision (Ferguson, 1989). A pronounced genetic differentiation has been reported both at the microgeographic scale (Ryman, 1983; Crozier and Ferguson, 1986; Bouza *et al.*, 1999) and along the geographic distribution range in this species. Five main phylogeographic lineages have been soundly identified using nuclear and mitochondrial DNA markers: Atlantic (AT), Mediterranean (ME), Adriatic (AD), Marmoratus (MA) and Danubian (DA) (Bernatchez, 2001; Presa *et al.*, 2002). A sixth lineage (Duero: DU) was recently identified, restricted to the Duero and Miño basins in the Atlantic slope of the Iberian Peninsula (Suárez *et al.*, 2001; Bouza *et al.*, 2008). These lineages exhibited a strong spatial partitioning and seemed to have evolved in allopatry with limited introgression among them, though some evidence of hybridization and introgression was revealed when studying the rDNA ITS1 (Internal Transcribed Spacer 1) (Presa *et al.*, 2002).

The Iberian Peninsula was one of the main glacial refuges during the Quaternary (Hewitt, 1996; Willis and Whittaker, 2000). The Duero basin, the largest river drainage in the Iberian Peninsula, drains to its Atlantic slope and shows specific biogeographic characteristics. Remarkable differences related with primary resources, slope, river flow and annual flow regime have been reported associated to northern and southern drainage areas (Arenillas Parra and Sáenz Ridruejo, 1987). A parapatric differentiation based on isozymes was reported in brown trout from Duero by Bouza *et al.* (2001). According to this information, two highly divergent genetic groups appeared segregated in northern and southern areas, with the highest divergence located at the Pisuerga tributary and the Lower-course (pure regions). However, a subsequent microsatellite and mtDNA survey in the same area suggested a slightly different scenario, with the same highly divergent pure regions, but with hybrid populations showing a more disperse and complex pattern (Martínez *et al.*, 2007). The location of the AT and DU mtDNA lineages, respectively, in the Lower-course and Pisuerga pure regions, suggested a secondary contact between both lineages after the last glaciation. The similar spatial segregation of AT and DU lineages in the Miño basin, a neighbour drainage located northwards, gives additional support to this hypothesis (Bouza *et al.*, 2008). Considering the time of divergence suggested for AT and DU lineages in the mid-upper Pleistocene (Bouza *et al.*, 2001; Suárez *et al.*, 2001) and the restriction of the DU lineage to NW Iberia, it is likely that other secondary contacts could have taken place in interglacial periods across the Quaternary (Martínez *et al.*, 2007).

Morphological characters are recommended for studying hybrid zones, especially in combination with genetic data (Campton, 1987; Barton and Hewitt, 1989). The analysis of morphological variation has been widely used by ichthyologists to differentiate species, lineages or populations within species (*e.g.*, Ihssen *et al.*, 1981; MacCrimmon and Claytor, 1985; Murta, 2000; Hermida *et al.*, 2005; Turan *et al.*, 2006). In brown trout, morphological, life-history or behavioural traits have sometimes demonstrated to be correlated with genetic differentiation (Ferguson and Mason, 1981; Giuffra *et al.*, 1994; 1996; Largiadèr and Scholl, 1996). However, some subspecies described based on morphological traits were attributed to environmental plasticity (Bernatchez, 2001). Morphological characters have the limitation of their polygenic basis and environmental influence. However, natural selection acts on the phenotype, and quantitative traits are essential to explain adaptation and evolutionary significant variation (Reed and Frankham, 2001). The lack or low correlation between neutral genetic markers, commonly used to identify genetic resources, and quantitative traits have advised to use both genetic and quantitative data to identify biological resources (Crandall *et al.*, 2000; Reed and Frankham, 2001; Cavers *et al.*, 2005). Such information facilitates management strategies for conserving biodiversity. The most suitable approach for analyzing morphological variation is the use of multivariate analysis on a wide set of morphometric and meristic traits. These multivariate methods have proven to be efficient tools for stock identification in management programs and for investigating taxonomic problems in sympatric populations of brown trout (Cawdery and Ferguson, 1988; Karakousis *et al.*, 1991; Aparicio *et al.*, 2005).

In the present work, brown trout populations from the Duero basin were studied using morphometric and meristic traits commonly applied in salmonids, to evaluate morphological variation in a secondary contact between divergent lineages. By contrasting morphological information with previous molecular findings we made an attempt to obtain a global and congruent explanation for the genetic segregation of highly divergent lineages of this species in the NW Iberian Peninsula.

**Figure 1:**
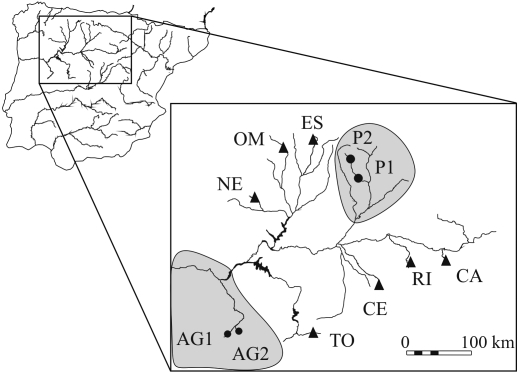
Location of the 11 sampling sites in the Duero basin (Iberian Peninsula) analysed in the present study. Pure samples (Pisuerga and Lower-course) are shown as solid circles in the grey areas and hybrid populations as solid triangles. Coordinates of the map: upper left border: 43°16' N/ 7°15' W; lower right border: 40°4' N/ 1°35' W.

**Figure 2:**
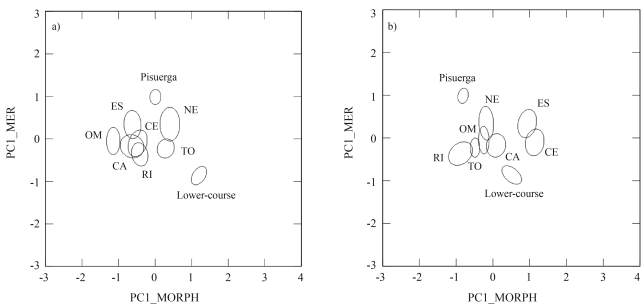
Representation of centroid plots (with 0.95 confidence ellipses) from the Duero populations using: a) first meristic principal component on first morphometric principal component, and b) first meristic on second morphometric principal component.

**Figure 3:**
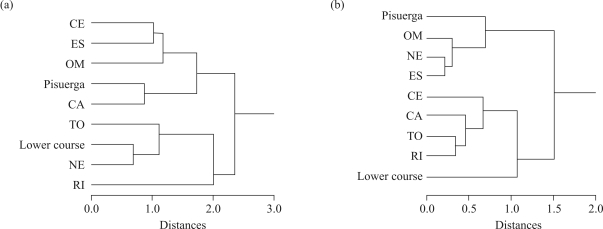
Cluster trees by Ward's method using Euclidean distances obtained for: a) hybrid populations using centroid functions as variables with morphometric traits and centroids calculated from pure samples, and b) all populations together using meristic traits and centroid functions as variables.

## Materials and Methods

### Sampling

Eleven samples of brown trout were collected by electro-fishing between autumn 2001 and spring 2002 from the Duero basin (Table 1; Figure 1). The sampling points were selected according to the following criteria: i) the native origin of the fish analyzed [both previous stocking and genetic information of sampling points were used to confirm the native condition of fish (Bouza *et al.*, 2001; Martínez *et al.*, 2007)]; ii) to obtain the most reliable picture of the morphological variation in this river basin, considering the main regions previously reported using different genetic markers (Bouza *et al.*, 2001: Martínez *et al.*, 2007). Accordingly, sampling included the pure regions (P1 and P2 from Pisuerga; AG1 and AG2 from the Lower-course) and hybrid populations (CA, CE, ES, NE, OM, TO and RI). These sampling points had been previously analyzed using isozymes, microsatellites and mtDNA. Sample codes are the same as in Martínez *et al.* (2007).

### Morphological analysis

Trout were sacrificed by an overdose of anaesthetic (ethylene glycol monophenyl ether). Twelve morphometric and four meristic characters were measured on fresh specimens shortly after death. The morphometric characters included caudal peduncle, body and head depth, eye diameter, distance between pectoral and pelvic fin, distance between pelvic and anal fin, maximum body width at the level of the dorsal fin origin, maximum gape width at the level of the posterior end of the maxilla, head and jaw lengths, and preorbital and postorbital distances. All measurements were taken to the nearest 0.01 mm, using a digital calliper. Before analysis, all morphometric measures were standardized according to the following expression:


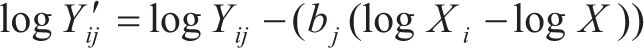


where *Y*_*ij*_' is the adjusted value of character j for individual *i*, *Y*_*ij*_ is the original value, *b*_*j*_ the allometric coefficient (the slope of the relationship between log *X*_*i*_ and log *Y*_*ij*_), *X*_*i*_ the standard length of individual *i*, and *X* a rounded-up mean of standard length from all samples (150 mm). The standard length is the length of fish measured from the tip of the snout to the posterior end of the midlateral portion of the hypural plate. Standardization of all morphometric measurements minimizes variability resulting from allometric growth and differences in mean size of individuals among populations (Reist, 1985). This correction is crucial in studies where the variation in mean size between samples due to different ecological features or biased sampling is relevant, as could be the case of the RI sample in our study.

The meristic counts included the number of gillrakers, pectoral and pelvic fin rays, and vertebrae. Counts of meristic bilateral traits were computed as the mean values from both body sides. All meristic traits were counted under a binocular microscope. The vertebrae were the last character counted, after scraping off muscle tissue. Separate analyses were conducted on the morphometric and meristic data, because these variables differ both statistically and biologically (Ihssen *et al.*, 1981). Sex was determined by visual inspection of the gonads.

A global estimation of divergence for each character between all populations was obtained by analysis of the variance. The mean relative interpopulation component of the variance was obtained both for morphometric and meristic traits. To analyze the cause of the global divergence, pairwise Tukey post-hoc tests were carried out for each character and population pair.

Principal component analysis (PCA) of variance-covariance matrix on morphometric and meristic variables was firstly used to reveal patterns of geographic variation between samples. For this analysis, the populations from Pisuerga (P1 and P2) and Lower-course (AG1 and AG2) were pooled, to obtain a single reference from each pure region. Components with eigenvalues > 1 were selected and scores of the loading matrix after varimax rotation evaluated. Centroid scores derived from the most informative principal components in each data set were labelled and plotted against.

Multiple discriminant analysis (MDA) was applied to the pure regions (Pisuerga and Lower-course) to get a discriminant function based on morphometric measurements. A classification matrix was constructed by assigning specimens to populations, on the basis of the linear combination of variables from the discriminant function. The jackknife method was used because it partially removes the bias inherent in classifying cases into groups that were used to define them (Tabachnick and Fidell, 1996). Then, this discriminant formula was used to compute canonical scores based on measurements from the hybrid samples, for comparing the values of pure regions with those of the hybrid populations. Diagram plots were used to display discriminant scores of each sample with respect to pure regions.

In addition, to assess the similarity between the hybrid populations, a second discriminant analysis was performed, using morphometric characters only from these populations. A classification matrix (jackknife method) was constructed by assigning specimens to populations, based on the linear combination of variables from each discriminant function. These discriminant functions were employed again to compute canonical scores, based on measurements from the two pure regions. A cluster analysis (Ward's method using Euclidean distance) was used to show the degree of similarity among the hybrid populations according to discriminant scores, using the group of centroids for each discriminant function as variables. Centroid groups from Lower-course and Pisuerga regions were added to the cluster analysis, to show their position with respect to other groups.

The same procedure described above was also applied to the meristic traits. However, the differentiation between pure regions was not so clear, and multivariate discriminant analysis was then applied to all populations together. As in the other cases, a classification matrix (jackknife method) was constructed and a cluster analysis (Ward's method using Euclidean distance) was used to show the degree of similarity among the different groups.

## Results

Analysis of the variance showed a highly significant divergence among the populations from the Duero basin (p < 0.001 for all characters except pelvic rays, p = 0.004). The relative component of variance was very high, ranging between 25.3 for pelvic rays and 60.7 for body depth (Table 2). The mean interpopulation component was 45.2 (S.D.: 10.8) and 31.5 (S.D.: 6.3) for morphometric and meristic traits, respectively, and 41.8 (S.D.: 11.5) when considering all characters. The results of the post-hoc tests revealed highly significant differences between all population pairs and characters (p < 0.001 tests: 51%; non-significant (NS) tests: 37.8%) that appeared evenly distributed across pairwise comparisons and traits. However, according to the number of significant test results obtained, three characters contributed in a higher proportion to the observed variation: head length: p < 0.001: 69.4%; NS: 25%; eye diameter: p < 0.001: 58.3%; NS: 25%; and body depth: p < 0.001: 63.9%; NS: 25%. Also, the Lower-course sample showed a stronger differentiation than the other samples (p < 0.001: 65.6%; NS: 22.9%). No significant differences between sexes were observed (ANOVA, p > 0.05 for all traits and samples). Main descriptive statistics for all morphometric and meristic traits can be found as Supplementary material (Table S1).

PCA on morphometric characters extracted three factors explaining 64.3% of the variance in the data (36.5%, 16.9% and 10.8%, respectively). The first component was related with several measures of the head (head depth, eye diameter, head and jaw lengths, preorbital and postorbital distance), whereas the second one was associated with traits related to body shape (caudal peduncle depth, body depth and body width) (Table 3). These two components showed no correlation with size (standard length; r = -0.04, p = 0.32; and r = -0.02, p = 0.62, respectively). The meristic counts were summarized in two principal components, accounting for 34.9% and 26.0% of explained variance, respectively. The number of gillrakers and pectoral fin rays was associated with the first component and the number of vertebrae and pelvic rays with the second one (Table 3). Plotting meristic PC1 on morphometric PC1 and PC2 evidenced a large heterogeneity among populations in the Duero basin (Figure 2). Pisuerga and Lower-course (pure regions) were the samples with the highest divergence when considering both plots. Some hybrid population pairs, like RI-ES or RI-CE, evidenced a similar divergence when plotting PC1-meristic against PC2-morphometric components (Figure 2b), but this divergence was much lower in the first plot (Figure 2a). The divergence between pure samples was mainly due to the meristic PC1, although both morphometric components revealed notable differences between the pure samples. The Lower-course sample showed a remarkable separation from the other ones, when comparing both PC1 components. As PCA does not require *a priori* grouping of the data, this assumption-free ordination of data justified the starting point hypothesis in the MDA analysis, to say that the pure samples constituted the extremes of the morphological range in the Duero basin.

The MDA using morphometric traits provided an almost complete segregation between the Lower-course and the Pisuerga samples, showing a clear-cut difference between the discriminant scores (Lower-course mean: 1.56, S.D.: 1.05; Pisuerga mean: -1.56, S.D.: 0.95). The most salient traits to discriminate pure samples were jaw length, head length and head depth (Table 3). Application of this discriminant function yielded a percentage of 94.1% of individuals correctly classified to their sample using the jackknife procedure. The discriminant scores computed for hybrid populations showed intermediate values between the pure regions in five out of seven samples (TO, mean: 0.22, S.D.: 1.11; CE, mean: 0.13, S.D.: 1.26; RI, mean: -0.29, S.D.: 1.38; NE, mean: -0.09, S.D.: 1.02; ES, mean: -0.29, S.D.: 1.22). The OM (mean: -1.66, S.D.: 0.92) and specially the CA population (mean: -1.84, S.D.: 1.23) displayed values beyond the discriminant function of the Pisuerga sample. The mean discriminant value for all hybrid samples was -0.546, clearly biased toward the score observed in the Pisuerga sample.

When the discriminant analysis was applied to the morphometric variables in the seven hybrid populations, six statistically significant discriminant functions (p < 0.001 in all cases) were found (Table 3). The first, second and third discriminant functions contributed to 40.8, 26.5 and 13.3% of the variance, respectively. The first function was mainly associated with body depth and eye diameter, the second one was related with head length and distance between pectoral and pelvic fin, and in the third function caudal peduncle depth and body depth were the most important variables. The application of discriminant functions yielded a percentage of 79.0% of individuals correctly classified to their respective population using the jackknife procedure (Table 4). The cluster tree obtained from Euclidean distances between population centroids including the scores of the pure regions showed two main groups (Figure 3a). The Pisuerga and Lower-course appeared clustered in different groups, and the hybrid populations were associated to the Pisuerga (ES, OM, CA, CE) and Lower-course (NE, RI, TO) groups without a particular geographic trend.

Four discriminant functions were obtained from the meristic characters, three of which were statistically significant (p < 0.001; Table 3). Each one of the four traits appeared associated mainly with one function, though the number of gillrakers was also related with the first function. The pectoral rays trait was related with the first function (60.6% of explained variance), vertebrae with the second (29.1%), number of gillrakers with the third (8.8%), and pelvic rays with the fourth (1.5%). With the meristic traits, 41.1% specimens were correctly classified to their respective populations using the jackknife procedure. The dendrogram with discriminant functions as variables using meristic traits showed a remarkable geographic trend, not observed in the morphometric one (Figure 3b). As in the first analysis, the Pisuerga and Lower-course samples were clustered in different groups, but the hybrid populations from the right margin (from upper to lower course) (OM, NE, ES) were associated to Pisuerga and those from the left margin (CE, TO, RI, CA) to Lower-course.

## Discussion

The large and significant differentiation among populations observed for all morphological traits studied in brown trout from the Duero basin is in accordance with the high genetic diversity detected previously with isozymes, mtDNA and microsatellites (Bouza *et al.*, 2001; Martínez *et al.*, 2007). The interpopulation component of the variance (all characters: 41.80%; morphometric traits: 45.22%; meristic traits: 31.52%) was very similar to the relative genetic differentiation component observed for isozymes (G_st_ = 0.46) and microsatellites (G_st_ = 0.348; R_st_ = 0.413). This differentiation was high across all population pairs and characters, as evidenced by the post-hoc tests performed. However, three characters (head length, eye diameter and body depth) showed a higher contribution to the global differentiation observed. This was in accordance with the high discrimination power of these characters evidenced by multivariate analyses. Likewise, the high percentages of individuals correctly classified to their respective samples after the application of discriminant analyses reflected the high morphological differentiation among populations in the Duero basin, as reported using the microsatellite markers (Martínez *et al.*, 2007). Though the hybrid populations did not show such a strong differentiation as the pure ones (94.1% classification success), they also displayed large heterogeneity (79.0% classification success using morphometric traits). Even when meristic characters were used, which involved only four traits with low variation, the classification matrix was not disappointed (41.1%). Finally, it is necessary to keep in mind that the influence of environmental variables on morphological traits (*e.g.* differences in temperature and oxygen content during early ontogeny) may contribute to the large variation observed.

The multivariate approaches enabled a more detailed analysis for finding out geographic trends in the whole differentiation observed in the Duero basin. All multivariate (principal component: PCA; discriminant: MDA) analyses and clustering methods consistently showed the highest morphological divergence between the pure regions, Pisuerga and Lower-course, as previously reported with molecular markers (Bouza *et al.*, 2001; Martínez *et al.*, 2007). This differentiation was mainly due to the meristic characters correlated with the first PC1 component, to say the number of pectoral rays and number of gillrakers. The hybrid populations displayed intermediate values for the MDA discriminant scores, except the CA and OM populations, which showed a mean discriminant value beyond the Pisuerga region. The CA population had also evidenced a hybrid value (admixture proportions; Bertorelle and Excoffier, 1998) beyond the Pisuerga region using microsatellite data (Martínez *et al.*, 2007). The hybrid populations showed discriminant values (mean: -0.546) closer to the Pisuerga (-1.560) than to the Lower-course region (1.560). This is in accordance with the previous microsatellite and mtDNA information (Martínez *et al.*, 2007), which evidenced a more relevant presence of the Pisuerga lineage in the Duero basin than previously reported in the study with allozymes (Bouza *et al.*, 2001). Finally, the Lower-course region appeared as the most highly differentiated sample in the PCA analysis, as had also been observed with microsatellite markers (Martínez *et al.*, 2007).

The results obtained demonstrate that the genetic differentiation reported in the Duero basin is associated to morphological variation. Morphological differences in brown trout have sometimes been associated to environmental plasticity (Bernatchez, 2001), but in other cases a correlation between both types of variation has been observed (Ferguson and Mason, 1981; Giuffra *et al.*, 1994; 1996; Largiadèr and Scholl, 1996). Our data provide insights on the main discrimination characters that could be related with specific adaptations to the environmental heterogeneity reported in the Duero basin (Arenillas-Parra and Sáenz-Ridruejo, 1987). The main morphometric divergence between the pure regions was related to head characters, which were highly correlated with the first principal component and the discriminant function. Several traits of head shape (*e.g.* length of jaw, mouth position or eye diameter) have demonstrated to be related to prey-detecting and handing in different species of fish (Cawdery and Ferguson, 1988; Ostbye *et al.*, 2005). According to the loading matrix, fish from Lower-course had longer jaws and heads and deeper heads than those from Pisuerga. Also, the relevance of some body shape variables (body depth, body width and caudal peduncle depth), both in the PCA and MDA analyses, indicate a more robust constitution of the Lower-course trout. Morphology has demonstrated to affect swimming ability in salmonids, when comparing different species, different populations or even migrating and non-migrating forms (Beacham, 1985; Ojanguren and Braña, 2003). In our study, the differences in meristic traits were mainly due to the number of pectoral rays, as evidenced by the PCA and MDA analyses. Salmonid juveniles have been suggested to use the pectoral fins to stabilize themselves when swimming against fast flowing water, so fins are useful to control propulsion (Ojanguren and Braña, 2003). All these differences could reflect adaptations to variable feeding and water-flow regime associated to the northern and southern areas of the Duero basin (Arenillas Parra and Sáenz Ridruejo, 1987).

The morphological variation found in our study is concordant with previous genetic data (Bouza *et al.*, 2001; Martínez *et al.*, 2007), confirming the spatial partitioning of brown trout from the Duero basin after a secondary contact between divergent lineages. The morphological traits responsible for this differentiation could be related with adaptation to the variable feeding and water-flow regimes across this large river basin. Selection in a highly heterogeneous environment could explain the restriction of the DU lineage to the inner part of the Duero basin along most of the Pleistocene.

## Figures and Tables

**Table 1:** Codes and sampling characteristics of the 11 brown trout populations from the Duero basin.

Code	Tributary	Region	Sample size
AG1	Águeda	Lower-course	51
AG2	Águeda	Lower-course	50
CA	Caracena	Hybrid population	41
CE	Cega	Hybrid population	51
RI	Riaza	Hybrid population	40
TO	Tormes	Hybrid population	52
ES	Esla	Hybrid population	50
OM	Orbigo	Hybrid population	46
NE	Negro	Hybrid population	34
P1	Pisuerga	Pisuerga	52
P2	Pisuerga	Pisuerga	49

Total			516

**Table 2:** Analysis of the variance of morphometric and meristic traits in the 11 populations from the Duero basin. Between- and within-populations absolute components and relative (%) between-population components are presented.

	Between populations	Within populations	% relative between pop.	F	p
Morphometric traits					
Body depth	3.613	2.339	60.70	76.860	< 0.001
Body width	0.925	1.312	41.35	34.727	< 0.001
Caudal peduncle depth	0.409	0.340	54.61	58.566	< 0.001
Eye diameter	0.200	0.150	57.14	61.010	< 0.001
Gape width	0.404	0.610	39.84	32.154	< 0.001
Head depth	0.494	0.769	39.11	31.761	< 0.001
Head length	1.763	1.416	46.78	58.813	< 0.001
Jaw length	1.178	0.821	58.93	71.45	< 0.001
Pectoral-pelvic fin distance	1.216	2.430	33.35	23.286	< 0.001
Pelvic-anal fin distance	1.850	2.408	43.45	33.737	< 0.001
Postorbital distance	0.546	0.754	42.00	33.830	< 0.001
Preorbital distance	0.144	0.421	25.49	17.991	< 0.001

Meristic traits					
Gillrakers	0.658	1.061	38.28	31.343	< 0.001
Pectoral rays	0.115	0.210	35.38	28.151	< 0.001
Pelvic rays	1.082	3.199	25.27	2.593	0.004
Vertebrae	0.182	0.488	27.16	18.556	< 0.001

**Table 3:** Structure matrix of discriminant loadings and loadings from principal component analysis based on morphometric and meristic traits in *Salmo trutta* populations from the Duero basin. MDA and PCA coefficients above 0.500 or below -0.500 appear highlighted in bold.

	MDA pure samples	MDA hybrid samples							PCA all samples		
Morphometric:	Can 1	Can 1	Can 2	Can 3	Can 4	Can 5	Can 6		PC 1	PC 2	PC 3
Body depth	0.479	**-0.643**	-0.094	**0.569**	0.182	-0.162	0.078		0.050	**0.900**	0.038
Body width	0.399	-0.388	0.079	0.118	-0.003	-0.145	0.367		-0.023	**0.837**	0.186
Caudal peduncule depth	0.423	-0.294	0.158	**0.620**	-0.226	0.367	-0.113		0.186	**0.819**	-0.142
Eye diameter	0.380	**0.581**	0.415	0.453	0.114	-0.030	0.009		**0.707**	-0.135	-0.418
Gape width	0.323	-0.373	0.291	0.142	**0.567**	-0.047	0.371		0.326	0.454	0.481
Head depth	**0.586**	-0.158	0.342	0.192	-0.072	0.006	**0.884**		**0.506**	0.390	0.270
Head length	**0.512**	-0.017	**0.595**	0.392	0.136	-0.446	-0.025		**0.880**	0.222	0.195
Jaw length	**0.660**	0.129	0.228	0.323	0.449	-0.009	0.136		**0.861**	0.124	0.115
Pectoral-pelvic fin dist.	-0.117	-0.249	**0.500**	-0.059	0.189	0.411	-0.197		0.061	0.033	**0.651**
Pelvic-anal fin distance	0.117	0.375	-0.354	0.315	-0.068	0.066	0.111		-0.158	-0.017	**-0.683**
Postorbital distance	0.403	-0.218	0.445	0.408	0.064	-0.284	-0.024		**0.648**	0.471	0.232
Preorbital distance	0.249	0.022	0.110	-0.203	0.009	-0.245	0.317		**0.600**	-0.083	0.326
		MDA all samples							PCA all samples		
Meristic:		Can 1	Can 2	Can 3	Can 4				PC 1	PC 2	
Gillrakers		**0.657**	-0.304	**0.661**	0.196				**0.761**	-0.002	
Pelvic rays		0.042	-0.047	-0.289	**0.955**				0.309	**-0.593**	
Pectoral rays		**0.834**	0.029	**-0.551**	-0.029				**0.825**	-0.050	
Vertebrae		0.149	**0.948**	0.268	0.090				0.202	**0.828**	

**Table 4:** Classification matrix applying the discriminant morphometric functions to the seven hybrid populations from the Duero basin. Population codes from Table 1.

Original group	Predicted group membership (%)							Total
	TO	CE	RI	CA	NE	ES	OM	
TO	86.5	0.0	1.9	1.9	7.7	0.0	1.9	100
CE	2.0	66.7	0.0	11.8	5.9	7.8	5.9	100
RI	0.0	0.0	85.0	0.0	5.0	2.5	7.5	100
CA	0.0	9.8	0.0	73.2	0.0	4.9	12.2	100
NE	0.0	2.9	0.0	0.0	97.1	0.0	0.0	100
ES	4.0	8.0	0.0	4.0	2.0	66.0	16.0	100
OM	2.2	4.3	0.0	0.0	0.0	8.7	84.8	100

Total corrected classified cases: 79.0%

## Supplementary Material

The following online material is available for this article:

Table S1 Number of individuals, mean, variance, standard deviation and range for the 12 morphometric and four meristic traits analysed in the nine samples from the Duero basin. The two Lower-course (AG1 and AG2) and Pisuerga (P1 and P2) populations were pooled in a single Lower-course (AG) and Pisuerga (P) sample (see Material and Methods).
