# Supplementary material for: Morphological variation in a secondary contact between divergent lineages of brown trout (Salmo trutta) from the Iberian Peninsula
Source: Genet Mol Biol. 2009 Jan 16;32(1):42–50. doi: 10.1590/S1415-47572009005000014 (PMC3032971; doi:10.1590/S1415-47572009005000014)
Supplement: Table S1 — Number of individuals, mean, variance, standard deviation and range for the 12 morphometric and four meristic traits analysed in the nine samples from the Duero basin. The two Lower-course (AG1 and AG2) and Pisuerga (P1 and P2) populations were pooled in a single Lower-course (AG) and Pisuerga (P) sample (see Material and Methods). [file gmb-32-1-42-suppl1.pdf]

Table S1 - Number of individuals, mean, variance, standard deviation and range for the 12 morphometric and 4 meristic traits analysed in the 9 samples from the Duero basin. The two Lower-course (AG1 and AG2) and Pisuerga (P1 and P2) populations were pooled in a single Lower-course (AG) and Pisuerga (P) sample (see Material and Methods).

Sample	Statistics	Trait								
		Standard Length	Body depth	Body width	Caudal peduncle depth	Eye diameter	Gape width	Head depth	Head length	Jaw length
AG	N	101	101	101	101	101	101	101	101	101
	Mean	109.8	24.0	12.8	10.7	7.6	9.7	14.3	29.2	14.2
	S.D.	42.3	9.7	5.1	4.0	2.0	4.1	5.3	11.4	6.1
	Variance	1791.2	93.5	25.9	16.0	4.0	16.9	27.7	129.9	36.9
	Minimum	49.3	10.8	5.5	5.0	4.4	4.1	6.9	12.8	6.0
	Maximum	205.5	47.2	25.8	20.2	11.4	21.3	27.2	56.3	29.2
CA	N	41	41	41	41	41	41	41	41	41
	Mean	129.6	28.3	15.4	11.9	7.3	11.5	15.3	31.6	14.2
	S.D.	79.4	18.0	9.2	7.4	3.5	7.2	8.5	18.5	8.9
	Variance	6309.8	325.5	84.5	55.5	12.0	51.5	72.5	342.6	78.3
	Minimum	46.7	9.1	5.4	4.2	3.4	4.2	6.3	12.3	5.0
	Maximum	245.2	59.1	29.5	22.9	12.1	25.6	30.5	59.9	30.3
CE	N	51	51	51	51	51	51	51	51	51
	Mean	116.4	27.2	14.3	11.8	7.3	10.7	14.6	29.2	13.4
	S.D.	39.3	9.8	5.1	3.9	1.7	3.8	4.5	9.3	4.7
	Variance	1545.1	96.0	25.9	15.4	3.0	14.5	20.0	85.9	21.9
	Minimum	51.4	10.6	6.6	5.1	4.2	5.1	6.7	13.6	5.6
	Maximum	196.5	47.6	24.8	19.1	10.5	18.8	23.1	48.2	23.8
RI	N	40	40	40	40	40	40	40	40	40
	Mean	62.2	11.8	6.6	5.6	5.2	5.4	8.3	16.8	7.4
	S.D.	14.4	3.1	1.6	1.3	0.8	1.1	1.6	3.3	1.6
	Variance	207.2	9.4	2.5	1.8	0.7	1.3	2.5	10.6	2.6
	Minimum	43.8	7.6	4.0	4.0	3.9	3.9	5.8	12.7	5.0
	Maximum	101.1	19.7	10.4	9.1	7.1	8.7	12.1	25.6	11.3
TO	N	52	52	52	52	52	52	52	52	52
	Mean	126.1	25.8	14.4	12.2	8.5	10.7	16.1	32.2	14.5
	S.D.	30.0	6.3	3.6	3.0	1.4	2.8	3.6	7.2	3.6
	Variance	898.5	40.3	12.6	9.2	2.0	8.0	13.1	52.0	13.1
	Minimum	84.0	16.5	9.7	7.7	6.7	7.2	11.4	22.4	9.9
	Maximum	208.7	40.7	23.2	19.1	11.5	18.7	25.6	49.5	23.0

Sample	Statistics	Trait								
		Pectoral-pelvic fin distance	Pelvic-anal fin distance	Postorbital distance	Preorbital distance		Pectoral rays	Pelvic rays	Gillrakers	Vertebrae
ES	N	50	50	50	50	50	50	50	50	50
	Mean	160.7	37.4	19.7	15.9	8.9	13.5	19.3	38.8	17.1
	S.D.	45.3	10.9	5.5	4.4	1.7	3.8	4.6	9.9	4.6
	Variance	2047.9	119.2	30.7	19.1	2.9	14.4	21.3	98.2	21.5
	Minimum	84.2	18.3	10.3	8.2	5.6	8.1	12.6	21.3	9.1
	Maximum	247.5	57.8	30.3	24.6	12.5	21.2	30.0	57.1	26.6
OM	N	46	46	46	46	46	46	46	46	46
	Mean	129.1	27.2	14.5	12.5	7.8	10.2	14.9	30.6	13.7
	S.D.	40.7	9.9	4.8	4.1	1.5	3.2	3.8	8.5	4.0
	Variance	1656.1	98.0	23.4	16.6	2.3	10.2	14.7	72.2	15.8
	Minimum	58.1	10.8	6.7	5.2	4.7	4.5	7.8	14.9	6,3
	Maximum	236.5	53.6	27.8	22.6	11.4	19.0	24.9	51.2	23,5
NE	N	34	34	34	34	34	34	34	34	34
	Mean	156.3	33.9	17.4	15.3	10.1	13.4	18.3	40.2	18,4
	S.D.	35.7	8.6	4.1	3.8	1.9	3.9	4.1	9.4	4,8
	Variance	1271.9	73.5	16.5	14.3	3.5	15.1	17.1	88.9	23,4
	Minimum	93.6	19.0	10.0	8.9	6.5	6.6	10.3	23.0	9,9
	Maximum	225.5	52.7	26.4	23.6	13.8	21.6	27.5	61.9	30,1
P	N	101	101	101	101	101	101	101	101	101
	Mean	136.8	27.9	14.9	12.6	8.3	11.1	16.0	34.1	15,9
	S.D.	27.3	6.3	3.1	2.5	1.2	2.3	2.8	6.4	3,4
	Variance	746.0	39.4	9.9	6.4	1.4	5.1	7.9	40.7	11,6
	Minimum	67.3	12.6	7.0	6.2	5.0	5.7	8.3	17.1	7,3
	Maximum	210.3	44.6	22.9	18.6	11.1	17.3	22.9	49.3	24,9
AG	N	101	101	101	101		101	101	101	101
	Mean	34.4	22.5	15.5	6.9		12.8	9.0	21.7	57.1
	S.D.	14.2	8.3	6.4	3.2		0.5	0.2	1.5	0.7
	Variance	202.6	69.1	40.5	10.3		0.2	0.0	2.2	0.5
	Minimum	14.0	9.4	6.6	2.4		12.0	8.5	10.5	55.0
	Maximum	69.9	40.2	30.6	15.8		14.0	9.5	24.0	59.0
CA	N	41	41	41	41		41	41	41	41
	Mean	41.2	26.0	17.2	8.0		13.2	9.0	22.0	58.5
	S.D.	25.5	16.5	10.4	5.4		0.4	0.2	1.1	0.5
	Variance	651.2	270.9	107.5	29.1		0.2	0.0	1.1	0.3

Sample	Statistics	Trait							
		Pectoral-pelvic fin distance	Pelvic-anal fin distance	Postorbital distance	Preorbital distance	Pectoral rays	Pelvic rays	Gillrakers	Vertebrae
	Minimum	15.0	8.7	6.7	2.2	12.5	8.5	20.0	58.0
	Maximum	77.1	51.2	33.5	18.8	14.0	9.0	24.0	59.0
CE	N	51	51	51	51	51	51	51	51
	Mean	37.0	24.3	16.0	6.6	13.4	9.1	21.6	58.1
	S.D.	13.4	8.7	5.5	2.4	0.5	0.3	1.1	0.8
	Variance	179.9	75.8	30.3	5.7	0.3	0.1	1.2	0.7
	Minimum	16.2	9.6	6.8	3.0	12.0	8.5	19.0	56.0
	Maximum	64.4	44.0	28.6	12.2	14.0	10.0	24.0	60.0
RI	N	40	40	40	40	40	39	40	40
	Mean	18.9	12.6	8.6	4.0	13.1	8.9	22.1	58.0
	S.D.	4.3	3.5	1.8	0.9	0.4	0.2	0.9	0.7
	Variance	18.4	12.1	3.2	0.8	0.2	0.0	0.9	0.5
	Minimum	13.8	7.3	6.3	2.9	12.0	8.0	20.0	56.0
	Maximum	31.0	22.0	14.0	6.3	14.0	9.5	24.0	59.0
TO	N	52	52	52	52	52	52	52	52
	Mean	40.4	26.4	17.1	7.5	13.0	9.0	22.5	58.1
	S.D.	9.9	6.7	4.3	1.9	0.4	0.1	1.2	0.6
	Variance	98.2	44.9	18.7	3.5	0.1	0.0	1.3	0.4
	Minimum	27.2	17.6	11.1	5.3	12.0	9.0	20.0	57.0
	Maximum	66.9	47.7	28.1	12.0	14.0	9.5	26.0	59.0
ES	N	50	50	50	50	50	50	50	50
	Mean	49.5	34.4	21.6	9.4	13.4	9.0	23.0	57.5
	S.D.	14.3	10.3	5.9	2.7	0.6	0.1	1.2	1.0
	Variance	205.2	105.2	34.6	7.4	0.3	0.0	1.5	0.9
	Minimum	25.8	16.1	11.7	4.6	12.0	9.0	21.0	53.0
	Maximum	78.7	53.6	34.0	16.4	14.0	10.0	25.5	59.0
OM	N	46	46	46	46	46	46	46	46
	Mean	40.1	27.7	16.6	7.3	13.3	9.0	22.4	57.6
	S.D.	13.4	9.0	5.1	2.3	0.5	0.1	1.1	0.5
	Variance	180.8	81.5	25.8	5.1	0.2	0.0	1.3	0.3
	Minimum	17.9	11,8	7.2	3.1	12.0	9,0	19.0	57.0
	Maximum	79.5	51.8	29.2	12.9	14,0	9.5	24.5	59,0

Sample	Statistics	Trait							
		Pectoral-pelvic fin distance	Pelvic-anal fin distance	Postorbital distance	Preorbital distance	Pectoral rays	Pelvic rays	Gillrakers	Vertebrae
NE	N	34	34	34	34	34	34	34	34
	Mean	48.6	33.8	21.8	9.1	13.5	9.0	22.7	57.7
	S.D.	11,3	7.5	5.4	2.3	0.5	0.2	1.0	0.8
	Variance	126,6	55.8	29.4	5.4	0.3	0.0	0.9	0.6
	Minimum	28.6	20.4	11.8	5.4	12.5	9.0	21.0	56.0
	Maximum	70,7	51.4	34.5	15.0	14,5	10.0	25.0	59.0
P	N	101	101	101	101	101	101	101	101
	Mean	43.3	27,2	18.3	8.0	13.8	9.0	23.6	57.6
	S.D.	9,0	6.0	3.6	1.9	0.4	0.2	1.3	0.7
	Variance	80.5	36,1	13.2	3.5	0.1	0.0	1.7	0.4
	Minimum	22,3	11.7	9.2	3.7	13.0	8.0	20.5	56.0
	Maximum	69.2	47.9	27.6	12.9	14.5	10.0	26.5	59.0
